# New Tools in HCV Diagnosis, in Light of the Enhanced Awareness and the New Drugs for Treatment: SMARTube and Stimmunology

**DOI:** 10.1155/2013/389780

**Published:** 2013-02-14

**Authors:** Svetlana Gorodin, Serhat Unal, Youchun Wang, Mikhail I. Mikhaylov, Ludmila Bigbulatova, Tamar Jehuda-Cohen

**Affiliations:** ^1^SMART Biotech Ltd., Rehovot 76705, Israel; ^2^Section of Infectious Diseases, Department of Medicine, Faculty of Medicine, Hacettepe University, Ankara, Turkey; ^3^Department of Cell Biology, National Institute for Control of Pharmaceutical and Biological Products, Beijing 100050, China; ^4^Chumakov Institute Poliomyelitis and Viral Encephalitides, Russian Academy of Medical Sciences, Moscow 142782, Russia; ^5^Laboratory Department, Kogalym City Hospital, Kogalym, Russia; ^6^Technion, Biomedical Faculty, Haifa, Israel

## Abstract

With improved HCV therapy, challenges regarding HCV diagnosis, such as seronegative window period, false positive readings, and differentiation between recent, chronic, and resolved infections, are of increasing importance. To address these challenges an innovative device—SMARTube HIV & HCV—was used. Blood samples were tested for anti-HCV antibodies before and after incubation in the SMARTube, which promotes the *in vitro* stimulation of *in vivo* HCV primed lymphocytes, thus enhancing levels of anti-HCV antibodies. Comparing antibody levels, in concordant samples before and after SMARTube, yielded the Stimulation Index (SI). Among 5888 fresh blood samples, from various populations and regions worldwide, 641 were seropositive using plasma, while SMARTube processing (yielding enriched plasma, termed SMARTplasma) enabled diagnosis of 10 additional carriers in high-risk cohorts, that is, earlier detection. Using SMARTplasma eliminated all false positive results, using the current assays. In addition we show that SI calculation may serve as an important tool for differentiating between those who recently seroconverted, carriers of long-term infection, and those who have cleared the virus. SMARTube and the SI could lead to better, more informative diagnosis of HCV infections and play an important role in changing the way we treat both the infected individuals and the epidemic as a whole.

## 1. Introduction

### 1.1. HCV Epidemiological Overview

 The hepatitis C virus (HCV) represents a global problem for public health systems worldwide due to its high prevalence, high rates of transfer, and severe health complications. Moreover, HCV is often missed during the initial stages of the disease due to lack of symptoms in the infected person [1, 2]. Later, most of these symptomless, yet HCV-infected, individuals will progress to chronic HCV infection [1, 3] and have a significant increased-risk of liver cirrhosis, hepatocellular carcinoma, and liver transplantation [3, 4].

 Hepatitis C is a global epidemic, and according to the World Health Organization (WHO), about 130–170 million people are chronically infected with hepatitis C virus worldwide [2]. Recent investigations have suggested that at least 5.2 million persons in USA [5] and over 5 million persons in the Russian Federation [6] are living with HCV today. It is estimated that each year 3-4 million people are newly infected with HCV, and more than 350,000 people die from hepatitis C related liver diseases annually [2]. HCV prevalence in the general population varies from 0,5% in Northern Europe, up to 2% in Mediterranean countries, ~3% in China, and above 4.8% in Pakistan, and reaches more than 20% in Egypt [2, 7–10].

 Incidence rates across the world vary and are difficult to calculate; however, information about the routes of HCV transmission [2, 11] and the populations with high risk for HCV infection [12] is important for estimating it. Recipients of blood, blood products, or organs [13–19], and injecting drug users (even those who injected drugs once many years ago) [20–22], are the highest risk groups in many countries. Patients on long-term hemodialysis also have a higher rate of HCV infection [23] than the general population. Prevalence of HCV infection among patients on hemodialysis therapy increases with the duration of hemodialysis treatment, and it varies between 5–70% [24–29]. It is also important to note the remarkably high occupational risk for health-care workers (HCWs). Hepatitis C virus is one of the most common blood-borne pathogens transmitted from patients to HCWs [30–34]. It is also transmitted tattooing, body piercing, and other forms of skin penetration. 

 According to CDC data [35], there are indications that sexual transmission of hepatitis C virus is associated with high-risk sexual activity and other STD's [36, 37]. Of note is also the fact that HCV viral loads are significantly elevated among individuals coinfected with HIV [38–40]. This also affects the rate of mother-to-child transmission which is estimated at 5%, while coinfection with HIV increases the risk to 19.4% [41]. 

In the last two years the HCV epidemic and its association with HIV have finally reached the international “spot light.” In order to increase the awareness and understanding of viral hepatitis and the diseases that it causes, the WHO has marked the 28th of July 2011 as the first official World Hepatitis Day [2]. CDC joined the WHO initiative, calling for a renewed commitment against a largely silent but persistent epidemic [42–44], hoping to provide an opportunity for coordinating a global worldwide response to the hepatitis epidemic. International awareness can help focus different efforts and resources on multifactor actions such as prevention, screening, and control of viral hepatitis and its related diseases. 

### 1.2. Challenges in HCV Management

In countries around the world (e.g., Canada, USA, Brazil, France, China, and Turkey), attention is focused on development and implementation of different approaches for management of hepatitis C virus (HCV) infection in order to diagnose the infection, guide treatment decisions, and assess the virological response to antiviral therapy [18–20, 45, 46]. 

New oral antiviral therapy for HCV [47–50], with lower toxicity and a shorter duration of treatment [51, 52], ushers in a new era. The advances in HCV treatment to make the arguments for early treatment much stronger, together with better tools for HCV diagnosis, could significantly improve treatment decisions [53].

There are two main categories of tests for HCV infection: tests for antibody and tests for viral antigens or viral RNA [54–56]. Detection of anti-HCV antibodies in plasma or serum is based on identification of IgG antibodies against several HCV antigens [57]. There are currently no assays for anti-HCV IgM antibodies. Thus on one hand, there are new effective drugs for early management of HCV infection while on the other hand, a diagnosis of early infection is often missed as the IgG anti-HCV antibodies are not detectable till several months after infection [48, 58–62]. Tools for differentiating between cleared and chronic infection are also needed [2]. 

The multiple challenges in HCV diagnosis can be divided into the following three groups: false negative test results during a long seronegative window period; significant and varying levels of false positive test results; differentiation between current/chronic infection and cleared/resolved infection. In addition, differentiation between recent and nonrecent infection would benefit both the patient (choice of treatment regime) and society (identification of sources of new infections).

#### 1.2.1. Negative Test Results during a Long Seronegative Window Period

Unlike most viral infections, antibodies against HCV do not appear within 5–10 days of the exposure to the infection. There is a seronegative window period (WP) of several months [58–65]. Which means that most of the recently infected individuals will test HCV seronegative during the WP and thus will be missed.

 The length of the WP depends, among other factors, on the general immune state of the patient [45] and may last as long as 6 to 12 months in immunocompromised or immunosuppressed patients [56, 66]. Immunosuppressive condition has been associated with HIV or HBV coinfection, organ transplants, and chronic hemodialysis [1, 56, 64, 66]. For example, there is a decrease of adaptive HCV-specific immune response in coinfection with HIV, [67]. Humoral immune responses to HCV during the acute phase were inhibited in the presence of active HBV replication, leading to poor antibody responses to HCV [68].

Antigen and nucleic acid amplification tests (NATs) allow for direct viral detection and have been shown to reduce the WP for detection of HCV infection by up to 60 days [69–71]. Due to high cost of NAT methods pooled samples (10–96 per pool) [60, 65, 72, 73] are used for donor screening in many of the developed countries [59, 72, 74–76]. Due to the pooling, the samples are diluted, leading to cases where pre-seroconversion donations were negative by both Ab testing and pooled PCR [72, 73, 75, 77, 78]. 

Kretzschmar et al. described a case of transfusion-acquired HCV infection from an extremely low-titer donation [77]. HCV transmission can be caused by donations that escape NAT detection even when tested in an individual donation. Several cases of hepatitis C transmission from HCV seronegative tissue donors have been recently reported in the USA. Four transplant recipients in Chicago have contracted HIV and hepatitis C virus from an organ donor who tested negative for both viruses [79]. According to US healthcare officials, that happened “because the donor was infected too recently for commonly used blood tests to detect the infection.” The United Network for Organ Sharing (UNOS) notified the US CDC of two patients who tested positive for HCV infection approximately 6 months after receiving kidney transplants from a seronegative, deceased donor [80]. This raised questions concerning current serologic testing policies and prompted a study to estimate the WP between infection and actual diagnosis by available laboratory testing algorithm [81]. 

In light of the above, finding a solution to the long WP remains an important goal in transfusion, transplantation, and diagnostic settings. Based on the fact that the long window period between HCV infection and detectable seroconversion is not due to lack of antigenic stimuli, it could be concluded that the WP is long, due to, at least in part, to specificimmune suppression [82]. Development of innovative technological solution which would overcome this immune suppression may lead to much needed progress in the field of earlier and better diagnosis of HCV infection. 

#### 1.2.2. Significant Level of False Positive Test Results

Current HCV diagnostic assays have high rates of false positives [83–85]. To add to the complexity of deriving true HCV prevalence information from testing programs and surveys, the false positive rates vary from population to population and from country to country [86–89]. This is a major concern as it means, on the epidemiological level, that we overestimate prevalence, in varying, and unknown, degrees, in different populations around the world. From another point of view, the low specificity of anti-HCV antibody assays [85, 88, 90] affects the blood supply, as it causes a loss of noninfected blood units, and the temporary deferral of the donor leads to additional potential loss of blood donations. 

#### 1.2.3. Problems Differentiating between Early/Recent Infection and Long-Term/Chronic Infection and the Need for Better Markers for Cleared or Resolved Infection

As high as 25% of HCV infected persons spontaneously clear the virus [1, 2, 91–95]. Current serology tools cannot differentiate between cleared/resolved infections and chronic/current ones. This is due to the fact that those who resolve the infection remain anti-HCV positive for many years [96, 97]. Clearance of hepatitis C depends on many factors such as genetic variants of host's genes [98], virus genotype [99], medical treatment of disease [100, 101], and general status of the immune system. Resolved infections have been frequently associated with a strong HCV-specific cellular immune response [89, 102, 103]. IgG has a half-life of 21 days [104], and therefore, following the clearance of the virus, high levels of anti-HCV antibodies will remain in the blood, for years to come. Using molecular assays for the detection of viral genome, as in the case of acute HCV infection, offers only a partial solution [97]. Moreover, lack of detection of HCV viral genome in the blood is not an indication of the state of HCV infection in the target organ (liver) [105, 106]. Thus, available assays do not provide effective tools for optimal differentiation of current and cleared HCV infection.

 These issues were in the spotlight of discussion during the 2011 HCV Symposium (CDC, Atlanta, USA, 2011). In particular, Dr. John W. Ward quoted the United States Department of Health and Human Services plan for the prevention, care, and treatment of viral hepatitis and emphasized the following actions as the most important: identify persons infected with viral hepatitis early in the course of their disease; assess new laboratory tests to accurately identify persons infected with viral hepatitis; develop and implement new technologies to improve viral hepatitis surveillance; develop approaches to detect and treat acute HCV in IDUs [53]. 

### 1.3. Stimmunology and the SMARTube as a Novel Solution to the HCV Diagnostic Challenges

A novel approach for improving anti-HCV antibodies-based diagnosis, which can overcome the challenges stated above, has been developed. The approach is based on Stimmunology, a technology which leads to the stimulation of antibody production, in a whole blood sample, *in vitro*, even in the face of specific immune suppression. Using this technology, via the SMARTube for HCV, enables us not only to look at the levels of HCV antibodies in the plasma, but also to measure the levels of HCV antibody produced *in vitro*, in a whole blood sample, thus measuring not only what has been produced in the past but also the current capability and potential for antibody production (or lack of) at the time of testing. 

 The innovative technology—Stimmunology (ST)—has been developed for the *in vitro* stimulation of *in vivo* immune-sensitized (or -primed) lymphocytes, specific to HIV, overcoming specific immune suppression and enhancing the production of HIV antibodies in culture. This was achieved by a proprietary formulation of bioactive compounds, in a tissue culture step. Using the Stimmunology process, the HCV-primed B cells, which are not yet producing anti-HCV antibodies *in vivo*, produce them *in vitro*, thus enabling the detection of the hepatitis C earlier in the course of the infection. This technology was initially developed for overcoming the WP in HIV (and SIV) infection. Using a monkey model of AIDS (SIV), it has been shown, in experimental infections, that the specific antibodies can be detected in the culture supernatant within days of infection, weeks and even months prior to seroconversion *in vivo* [107, 108]. Similar findings for overcoming the HIV seronegative window period were reported in studies where Stimmunology was used as a blood sample processing step, inside the SMARTube, prior to routine lab testing for HIV antibodies [109–113].

 Following the development of the SMARTube for HIV, a SMARTube for HCV was developed, and then the two formulations were combined to create a stimulation formula for both HIV and HCV in one SMARTube. Following its development the SMARTube was tested, as a blood pre-treatment step prior to HCV antibody testing, in various cohorts and populations across different geographical regions worldwide. The aim of the studies was the evaluation of SMARTube-based detection of HCV-infected individuals, as an improved tool for overcoming the multiple challenges of HCV diagnosis. 

## 2. Materials and Methods

In order to evaluate the efficacy of the SMARTube as a tool for solving some of the problems of HCV diagnosis, clinical trials were conducted in several populations with high or low risk for HCV, from geographical regions with different prevalence of HCV [116]. A total of almost six thousand blood samples, both with and without SMARTube pretreatment, were tested for anti-HCV antibodies. The fresh blood samples were collected in Israel, China, Romania, Kenya, Turkey, Hungary, and Russia during research and/or routine clinical testing over a period of over ten years (1997–2010). A list of specimens for analysis and cohorts studied are presented in Table 1. 

**Figure 1:**
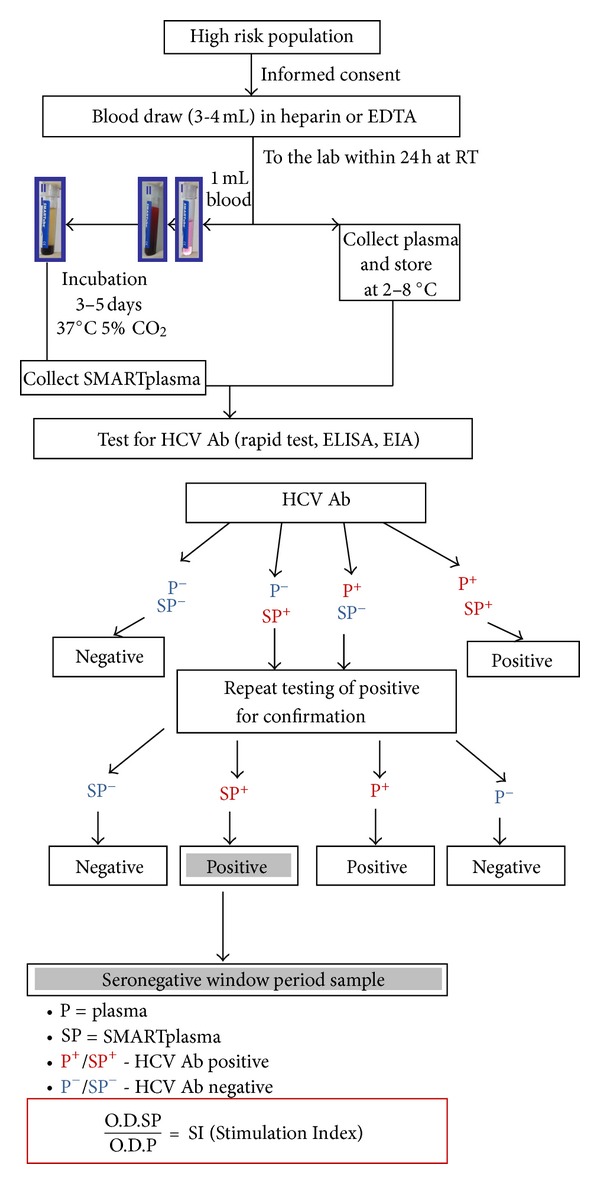
Clinical study flow chart.

 Blood samples, from listed populations, were collected into heparin containing tubes and kept at room temperature till laboratory handling within 24 hours. From each sample of heparinized whole blood, 1 mL was put into SMARTube HIV & HCV [117] for the Stimmunology processing. After 3–5 days of incubation in tissue culture conditions (at 37°C in a 5% CO_2_ humidified incubator), the supernatant, termed SMARTplasma, was collected for anti-HCV antibody testing (Figure 1). The testing of SMARTplasma was always done in parallel with the regular plasma samples, using standard serology assays and the routine diagnostic algorithm, as approved in the country of testing. Cut-off value was calculated according to manufacturer's instruction in each case separately. All initially reactive samples were confirmed by repeat testing of the same sample, on the same ELISA and/or on an additional different antibody tests. The ratio between the antibody levels in the SMARTplasma and the antibody levels in the parallel plasma (from the same blood sample), termed the Stimulation Index (SI), was calculated for each individual. 

 All clinical studies were conducted in conformation with the Helsinki agreements and local guidelines. The studies described involved only adults and were part of the general testing routine. Most of the samples were unlinked, and the blood samples were identified by a serial number only with no other identification details. Due to the clinical studies being cross-sectional, with most of the samples unlinked, follow-up testing was usually not possible.

## 3. Results and Discussion

### 3.1. HCV Prevalence in Studied Populations and Regions

A total of 5888 blood samples were tested by standard serology algorithm, and prevalence of anti-HCV antibodies in the target populations was calculated based on confirmed serology results (see Table 1). According to WHO and CDC data [2, 118], prevalence of HCV infection varies among various geographic regions. In our study, HCV prevalence varied from 5-6% in Mediterranean countries to 12–25% among patients from AIDS Centers in Eastern Europe. A rather high percentage of individuals with anti-HCV antibodies (18%) was found among those with a suspected recent exposure to hepatitis C in Romania. The highest prevalence (59.57%) was found in a cohort of intravenous drug users from Sichuan province, China. Regional prevalence rates in China were reported to range from 0% to 31.9% [118]. Most of the infections, both in China and in Eastern Europe, were attributed to transmissions during the 1980s and 1990s which were associated with transfusions of unscreened blood and injections with improperly sterilized equipment [119–124]. 

### 3.2. SMARTube Enabling the Detection of Window Period Samples

The blood for the Stimmunology step and the plasma were both from the same blood sample, and thus the resulting cultured supernatant, termed SMARTplasma, and the plasma samples could be compared (Table 2). Among the 5888 blood samples which were collected from various groups in different geographical regions, 641 were seropositive in routine serology testing. Seropositive samples were also positive following the SMARTube step; that is, no loss of diagnostic sensitivity was observed. 

 The SMARTube was used to enhance the *in vivo* primed lymphocytes, in a whole blood sample, to proliferate and differentiate, leading to *in vitro* stimulation of specific antibody synthesis. In the reported studies, the SMARTube led to the diagnosis of 10 additional positive persons, thus increasing the diagnostic sensitivity of the antibody assays used. No additional HCV positive samples were observed, using SMARTplasma, among blood donors and among the general population, across all observed geographical regions, indicating no loss in diagnostic specificity.

 Of 950 SMARTplasma, four and five window period samples were from individuals with high risk of HCV transmission and 40 from individuals who were recently exposed to HCV, respectively. All samples which tested positive only after the SMARTube incubation were confirmed by second HCV ELISA positive test of the SMARTplasma (data not shown).

 It should be noted that the level of increase in diagnostic sensitivity depends on both the length of the seronegative window period and the incidence level in each population. In the current study, the highest level of recent infections was observed among individuals who reported exposure to HCV in the last 1–3 weeks (8.1%). Seven of those 40 tested individuals were seropositive by regular serology, indicating nonrecent infections, and five were missed by regular serology and detected only using the SMARTube, indicating an infection which could be due to the reported exposure. Similar results were reported among individuals at high risk for HIV and in populations with a high incidence rate [125]. Such findings in the Ethiopian [110] and Kenyan [112] cohorts seem to indicate a longer window period for HIV in those African populations. 

 Thus, using the SMARTube overcomes the challenge of the long seronegative window period in HCV infection. Ability to detect infection earlier, without having to wait for the *in vivo* production of antibodies to reach detectable levels, could provide an opportunity for treatment by new drugs for early HCV therapy. Starting treatment at such early stages might enable, in the future, lower doses of drugs and shorter treatment duration [53].

### 3.3. ST as a Tool for Decreasing False Positive Rates in HCV Diagnostic

Among the 5888 blood samples tested using both plasma and SMARTplasma there were 673 initially reactive plasma samples (Table 3). Of these, 32 tested as clear negative using SMARTplasma as the sample. Repeat testing of those 32 initially reactive plasma samples showed them to be false positive readings, indicating that the SMARTplasma results were true, and that using the SMARTube reduced the false positive rate by 100%. The seven patients from Kogalym Hospital in Russia, who were initially antibody positive in plasma and SMARTplasma negative, were followed for 1 year, and none of them seroconverted. Of special concern is the high rate of false positive results among the initially reactive samples in low risk populations. Among 2810 healthy blood donors from different countries, there were 11 plasma positive donors while 7 (63.6%) of them were negative by SMARTplasma. These 7 were also negative upon repeat testing of the plasma, indicating that the negative results following Stimmunology pretreatment were all noninfected, bringing to zero the false positive rate. 

 Similar results were reported during the laboratory evaluation of SMARTube-enabled HIV detection among the replacement donors' population in Kenya. All samples which were plasma positive yet were SMARTplasma negative at the initial testing were found to be plasma negative upon repeat testing, that is, gave a false positive reading at the initial screening. Thus, in the diagnosis of both HCV and HIV infection, using the SMARTube, and testing the SMARTplasma, instead of plasma, has improved the specificity of the currently available antibody tests by as high as 100%. This is probably due to two processes which take place during the incubation of a blood sample in the SMARTube: the SMARTube preanalytical step leads to elevation of the specific signal, by increasing of HCV- and/or HIV-specific antibodies level in the blood sample; and the incubation of the whole blood in the SMARTube decreases the relative levels of nonspecific antibody binding and possible “noise” by the dilution of the blood sample (1 mL blood = ~0.5 mL plasma) by the SMARTube solution (2 mL).

 It is well known that there is a negative correlation between the levels of diagnostic sensitivity and specificity. Increasing diagnostic sensitivity usually comes at the account of reduced specificity and vice versa. This is true for diagnostic kits and assays; however, the SMARTube is not a diagnostic kit. While an increase in the diagnostic sensitivity, when using SMARTplasma versus plasma, could have been accompanied by a decrease in diagnostic specificity, this is not the case. The above-reported data indicate that the Stimmunology step actually decreased (actually eliminated, in these studies) the rates of false positive readings among blood donors and low risk groups; that is, it increased the specificity of the testing kits. Since, according to blood banks' safety rules, any blood unit with a positive antibody reading must not be released for clinical use [70, 74, 112], any false positive reading means a loss of a blood unit and some delay in reentry of the donor into the donor pool. In view of the ongoing shortage of donors, blood units and blood components, improving the specificity of available screening tests offers an opportunity to reduce the loss of good blood units, which has both economical and medical benefits [126].

### 3.4. Differentiating HCV Infection Stages by the Stimulation Index (SI) Parameter

The ability of the SMARTube HIV & HCV to drive forward HCV antibody production, in a whole blood sample, is dependent on the presence of lymphocytes, primed *in vivo* by hepatitis C virus's antigens. HCV-primed B cells (and T cells) would be present in the blood within days of infection, and newly produced naïve B cells will be primed by viral antigens for as long as the viral infection will persist [127]. Once the infection is cleared (spontaneously, or following ARV treatment), there will be no further priming of naïve B cells (or T cells). At that time, while the already produced antibody levels in the blood will remain high for several years, the ability to enhance further antibody production, *in vitro*, by newly primed B cells would be gone within days. This hypothesis leads to additional analysis of the studies' results. In order to give a quantitative expression of the described processes, the ratio between the antibody levels in the SMARTplasma and the antibody levels in the plasma—the Stimulation Index (SI)—was calculated for each individual. 

 According to the obtained clinical laboratory results analyzed with SI, allocation can be made for four different possible intervals of values for this parameter. Moreover, each interval of SI values could serve as an indicator of a different stage in the HCV infection disease course.

 Comparative analysis of HCV antibodies concentrations in SMARTplasma and plasma which were missed by regular serology, but detected after Stimmunology processing, shows very high “ratios” between antibody levels before and after Stimmunology blood pretreatment. Shortly after infection and prior to seroconversion, the antibody levels in the SMARTplasma would be much higher than in the plasma (which is actually negative, i.e., no (zero) OD due to specific antibodies), and thus the Stimulation Index is infinitesimal, that is, SI = ∞ would indicate very early infection, or in other words, the seronegative window period.

 Shortly after seroconversion there would be many HCV-primed B cells which have not yet matured to plasma cells. In our study, SI > 1.2 was considered to probably indicate recent seroconversion due to the fact that antibody production *in vivo* has not yet reached its full capacity.

 During the chronic infection, the difference between the antibody levels in the SMARTplasma and in the plasma is insignificant. This is the reason why an SI value around 1.0 (0.8 < SI < 1.2) indicates a long-term infection. 

 The decline of SMARTplasma antibody levels, which happens shortly after the clearance of the HCV infection, can be explained by the fact that the level of antibodies in the SMARTplasma is dependent on both the antibodies already present in the blood and the newly produced antibodies, in culture, by HCV-primed B cells. With the primed cells gone from the blood shortly after the clearance of the virus from the body, the *in vitro* production will be gone too. Thus, the decrease of antibody levels in the SMARTplasma will precede the one observed in the plasma, and an SI index in interval SI < 0.8 indicates a cleared infection. 

The data of the HCV positives from the different clinical studies was analyzed for its SI values,and the results are presented in Table 4. In the blood samples tested in Russia, which were positive for HCV (total 13 samples), all the infections seem to be in the chronic phase. No recent infections and no infections in the window period were found. With such a small sample set it is statistically expected that the more rare situations (e.g., recent infection) will not be found. The same is true for the WP samples—of which none were detected.

 In Hungary and Kenya there were ~12% who cleared the infection, according to SI calculation. While no samples were from recently seroconverted individuals, there was one sample from a person in the WP in each country. In contrast, in Romania, Turkey, and China, more than one sample were detected in WP or with recent HCV infection (5, 3, and 11, resp.), indicating a high incidence level in the studied populations. In epidemiological terms we can see how the SI could serve as a tool for incidences estimation. While in Romania and Turkey there were rather high (~30%) levels of cleared infections, there were none among the Chinese samples. The majority of the HCV-positive patients, in all studied populations, had a chronic infection. 

The presented data shows that SI calculation may be used as a tool for differentiating between different stages of the HCV infection. The SI information could enable the distinction of those who recently seroconverted form carriers of long-term infection. This parameter can lead to identification of a person infected with viral hepatitis, early in the course of the disease and treat acute HCV with the new therapeutic resources. In addition, the SI can indicate cases of cleared infection and patients who do not need treatment or their treatment has been effective. 

## 4. Conclusions

Identification of all HCV-infected individuals in high risk populations is important for early treatment, both for the sake of the patient and for society. Detection of all HCV infections among blood/organ donors could reduce the risk of HCV transmission caused by transfusion, transplantation, and dialysis. Using the innovative technology—Stimmunology, and its device, the SMARTube HIV & HCV, as a preanalytical step prior to testing for HCV antibodies—enables the detection of additional infected carriers—those who are in the window period. Detection of these routine seronegative yet infected individuals is impossible by the currently available assays. In addition, SMARTube pretreatment improves specificity of HCV diagnostic assays and allows reduction of false positive results. Thus, the SMARTube could be applicable for changing the way we treat both the infected individual and the epidemic as a whole.

## Figures and Tables

**Table 1:** Prevalence of HCV antibody positive individuals detected using regular plasma.

Risk of HCV transmission	Population/cohort studied	Country	Total samples tested	Serology positive**	HCV prevalence rate
Suspected HCV infection	Timisoara hospital patients, with suspected HCV infection [114]*	Romania	143	27	18.90%
	Testing of individuals, 1–3 weeks after reported suspected exposure to HCV	Romania	40	7	17.5%
	Hacettepe University Hospital patients with suspected HCV infection	Turkey	500	22	4.4%

Populations with high risk of HCV transmission	Seronegative individuals with very high risk of HCV transmission	Israel	67	4	5.97%
	Intravenous drug users, (Sichuan Province)	China	653	389	59.57%
	Discordant (HIV) couples—patients of Moscow AIDS Center [109]*	Russia	24	6	25%
	Patients of Budapest AIDS center [115]*	Hungary	206	25	12.13%

General populations with low risk behavior and unknown HCV prevalence	Ethiopian immigrants in Israel, unknown prevalence of HCV in population	Israel	238	3	1.26%
	Unknown prevalence of HCV in population (Hebei Province), infection due to bad medical practices in the past	China	583	139	23.84%
	Kogalym hospital patients and medical staff, population with mixed risk level	Russia	330	7	2.12%
	Replacement blood donors in Kenyatta blood bank, unknown prevalence	Kenya	294	8	2.72%

Low risk populations	Healthy blood donors (Tel Aviv)	Israel	625	0	0
	Healthy blood donors (Beijing)	China	1552	4	0.26%
	Healthy blood donors (Bucharest)	Romania	608	0	0
	Healthy blood donors (Moscow)	Russia	25	0	0

*Data was presented at a local conference.

**HCV seropositives, confirmed by local algorithms.

**Table 2:** Early HCV infections missed by current serology and detected using the SMARTube pretreatment step and SMARTplasma as the sample tested.

Population studied	Positive using plasma*	Positive using SMARTplasma	Individuals in window period**	Rate of missed infections***
Suspected HCV infection	56	61	5	8.1%
Populations with high risk of HCV transmission	424	428	4	0.9%
Regular populations with unknown HCV prevalence	157	158	1	0.6%
Healthy donors, low risk populations	4	4	0	0%

Total	641	651	10	

*HCV seropositives, confirmed by local algorithms.

**HCV infections missed by regular serology, but detected using the SMARTube HIV & HCV.

***Rate of missed infections was calculated as the % of HCV positive samples, which were in the window period, from the total infected individuals in that population.

**Table 3:** HCV false positive rates using regular plasma versus SMARTplasma as the tested sample.

Population studied	Total samples tested	Initially reactive plasma samples	False positive plasma samples*	False positive results rate in population**	False positive SMARTplasma samples
Suspected HCV infection	683	64	8	1.1 %	0
Populations with high risk of HCV transmission	950	431	7	0.7%	0
Regular populations with unknown HCV prevalence	1445	167	10	0.7%	0
Healthy donors, low risk populations	2810	11	7	0.2%	0

Total	5888	673	32	0.5%	0

*False positive by regular serology according to the local algorithms (e.g., negative upon repeat testing).

**Percent of false positive results among total plasma samples tested in that population.

**Table 4:** Stimulation Index (SI) distribution among HCV infection disease stages (according to clinical laboratory results of HCV antibody testing on current assays).

Countries	HCV infection stages and SI distribution			
	Window period* SI = ∞	Recent seroconversions** SI > 1.2	Chronic infections** 0.8 < SI < 1.2	Cleared infections** SI < 0.8
Russia	0	0	13	0
Romania	5	0	21	9
Turkey	0	3	12	7
Kenya	1	0	7	1
Hungary	1	0	19	4
China	2	9	520	0

*HCV infections missed by regular serology, but detected using the SMARTube blood pretreatment step.

**HCV seropositives, confirmed by local algorithms.

## Abbreviations

HCV: HepatitisCvirus
WHO: WorldHealthOrganization
ST: Stimmunology
SI: StimulationIndex
WP: Windowperiod
ELISA: Enzyme-linkedimmunosorbentassay
OD: Opticaldensity
IDU: Intravenousdrugusers.
